# Supplementary material for: Built structures influence patterns of energy demand and CO2 emissions across countries
Source: Nat Commun. 2023 Jul 3;14:3898. doi: 10.1038/s41467-023-39728-3 (PMC10317978; doi:10.1038/s41467-023-39728-3)
Supplement: Supplementary file 1 — Supplementary Information [file 41467_2023_39728_MOESM1_ESM.pdf]

SUPPLEMENTARY INFORMATION:

Built structures strongly influence cross-country patterns of energy demand and CO_2 emissions

Helmut Haberl[1,*,**], Markus Löw[1,**], Alejandro Perez-Laborda[2,**], Sarah Matej[1], Barbara Plank[1], Dominik Wiedenhofer[1], Felix Creutzig[3,4], Karl-Heinz Erb[1], Juan Antonio Duro[2]

[1] Institute of Social Ecology, University of Natural Resources and Life Sciences, Vienna

[2] Economics Department and Eco-SOS, Universitat Rovira i Virgili

[3] Mercator Research Institute on Global Commons and Climate Change, EUREF 19, 10829 Berlin

[4] Technical University Berlin, Straße des 17 Junis 135, 10623 Berlin

[*] corresponding author: helmut.haberl@boku.ac.at

[**] these authors contributed equally to this paper

Contents

This supplementary information is complementary to the Results and Methods sections of the main article and provides a documentation of the definitions and procedures applied. We document information on data sources and the definitions used in defining the material stock pattern indicators. We report on all specific steps taken, and also show additional maps and results of alternative statistical analyses.

Table of contents

1. Data sources 4

1.1 Spatial datasets 4

1.1.1 Choice of built-up land dataset 4

1.1.2 Dataset used to represent road and railway infrastructures 4

1.1.3 Data sources 5

1.2 Socioeconomic and socioecological datasets 6

2. Pre-processing of the spatial dataset 7

3. Spatial indicators 9

3.1 Built-up land indicators 11

3.1.1 Built-up land fraction (BL_{fract}) 11

3.1.2 Built-up land per capita (BL_{cap}) 11

3.1.3 Dispersion of built-up land (BL_{disp}) 11

3.1.4 Monocentricity of built-up land (BL_{mono}) 12

3.1.5 Compactness of built-up land (BL_{comp}) 12

3.1.6 Urban population density (UP_{dens}) 13

3.2 Road network indicators 13

3.2.1 Road density (RD_{total}) 13

3.2.2 Urban road density (RD_{urban}) ... 14

3.2.3 Rural road density (RD_{rural}) ... 14

3.2.4 Urban-rural road length ratio ($RL_{urb-rur}$) ... 14

3.2.5 Urban-rural road density ratio ($RD_{urb-rur}$) ... 14

3.3 Railway network indicators ... 15

3.3.1 Railway density (RWD_{total}) ... 15

3.3.2 Urban railway density (RWD_{urban}) ... 15

3.3.3 Rural railway density (RWD_{rural}) ... 15

3.3.4 Urban-rural railway length ratio ($RWL_{urb-rur}$) ... 15

3.3.5 Urban-rural railway density ratio ($RWD_{urb-rur}$) ... 16

4. Supplementary maps ... 16

5. Additional analyses ... 24

5.1 Bivariate analyses using indicators related to total territory ... 24

5.2 Alternative lasso approaches ... 25

5.3 Alternative variable section using forward stepwise regressions ... 29

Data and Code availability ... 31

Supplementary References ... 31

1. Data sources

1.1 Spatial datasets

1.1.1 Choice of built-up land dataset

Several built-up land datasets can be used to analyse spatial patterns of global settlements and infrastructures[1–3]. These datasets are limited to built-up land and do not contain data on other land-cover classes. These were required for calculating the 'inhabited land' layer, which we used as reference area for many indicators (see Methods, section "Reference layer for inhabited land"). We hence needed a validated and reliable land cover dataset with full global coverage. High validity and comprehensive coverage of the entire land surface was more important for our approach than high spatial resolution; hence we decided to use the official 100m land cover mapping product of 2015 (LC100). This product is provided by the European Space Agency via its long-term Copernicus Global Land Service[4–6]. The LC100 has an overall classification accuracy of 80.6%, assessed at more than 28.000 independent validation points[6]. Because this dataset is updated annually, it will enable researchers to embark on comparable studies in the future. A spatial resolution of 100m is sufficient for the purpose of this study because 100m are within walking distance and energy-intensive motorized movement is generally not required at that scale. Using data at a higher spatial resolution would have limited added informative value but needlessly raise processing time, as computing power rises exponentially with resolution.

1.1.2 Dataset used to represent road and railway infrastructures

Recent studies manually compiled national infrastructure data to create global road and railway datasets that are considered as being robust and verifiable; e.g. GRIP[7]. Meanwhile, crowd-sourced data in the Open Street Map (OSM) have emerged as a plausible alternative. Several studies have demonstrated that OSM can be used as reliable data source for mapping urban areas[8–10], and OSM road data have been shown to be ~80% complete at the global scale[11] and are widely used[12–16].

How homogenous such datasets are, depends on the hierarchical level of infrastructure features (e.g. from primary to tertiary roads). In datasets such as OSM, which source volunteered geographic information (VGI), data on higher-level features are more likely to be robust because they are visible to more contributors[17], whereas data quality is more heterogenous for minor roads, unpaved or bicycle paths[16]. While manually compiled datasets such as GRIP are plagued by problems resulting from inconsistent nomenclatures because the data originate from different sources, OSM data are collected using a harmonized nomenclature. Still, data quality related to the minor infrastructure network is variable also in OSM. Hence, we improve the comparability across countries and world regions by excluding minor road and railway features (Supplementary Table 3). We performed visible inspection

of 10 cross-border regions on all continents to filter the available OSM data, which revealed that the filtered data are substantially more complete and better comparable across space than manually compiled datasets such as GRIP (for an example, see Supplementary Figure 1). We hence think that the resulting network maps depicting major roads and railways are sufficiently robust and complete for the purposes of this global-scale study.

1.1.3 Data sources

For data preparation we used four global spatial datasets, comprising land cover, elevation, infra-structure, administrative and population data (Supplementary Table 1). Code used in data preparation is freely available online[18].

Supplementary Table 1. Spatial datasets used in this study

Name	Abbrev.	Type	Resolution	Provider	URL
LC 100m: Copernicus Global Land Service (2015)[4]	LC100	grid	100 m cell	ESA, Copernicus	https://zenodo.org/record/3243509#.XnswsmAxnIU
NOAA Digital Elevation Model (1999)[19]	NOAA-DEM	grid	1 km	NOAA	https://www.ngdc.noaa.gov/mgg/topo/globe.html
Open Street Map data (May 2020)[20]	OSM	vector	-	Geofabrik	https://download.geofabrik.de/
National territory borders (2016)[21]	NT	vector	national	EUROSTAT, GISCO	https://ec.europa.eu/eurostat/web/gisco/geodata/reference-data/administrative-units-statistical-units/countries

1.2 Socioeconomic and socioecological datasets

To complement the spatial indicators in Supplementary Table 1, we used several socioeconomic and socioecological indicators ('conventional factors') for the statistical analyses (Supplementary Table 2). Data used in the analysis are freely available online[22].

Supplementary Table 2. Socioeconomic and socioecological datasets used in the analysis

Name	Abbrev.	Years	Nr. countries	Provider	URL
Gross domestic product (constant 2015 prices, US$/yr)	GDP	2015-2019	212	United Nations	https://unstats.un.org/unsd/snaama/downloads
Population Data	NPD	2015-2019	208	World Bank	https://data.worldbank.org/indicator/SP.POP.TOTL
Urban population rate (% of total population)	UPOP	2015-2019	201	World Bank	https://data.worldbank.org/indicator/SP.URB.TOTL.IN.ZS
Pump price for gasoline (US$/liter)	PGAS	2012-2016	176	World Bank	https://data.worldbank.org/indicator/EP.PMP.SGAS.CD
Heating degree days (°C days, ref: 18°C)	HDD	2015-2019	204	IEA	https://www.iea.org/articles/weather-for-energy-tracker
Territorial CO_2 emissions (ktC/yr)	CO_2	2015-2019	205	GCP	https://www.icos-cp.eu/science-and-impact/global-carbon-budget/2020
Total final energy consumption (TJ/yr)	TFC	2013-2017	140	IEA	https://www.iea.org/data-and-statistics/data-product/world-energy-balances

2. Pre-processing of the spatial dataset

All procedures aimed at increasing homogeneity of the dataset across space and were applied separately for every country (see Section 1.1.2). In total we analyzed 203 countries. Inclusion or exclusion of road types is reported in Supplementary Table 3, those of railway types in Supplementary Table 4.

Supplementary Table 3. Road types of OpenStreetMap data[20]

OSM road types (R)		
name (fclass)	**osm_code**	**included [y,n]**
motorway	5111	y
trunk	5112	y
primary	5113	y
secondary	5114	y
tertiary	5115	y
unclassified	5121	y
residential	5122	y
living_street	5123	y
pedestrian	5124	y
motorway_link	5131	y
trunk_link	5132	y
primary_link	5133	y
secondary_link	5134	y
tertiary_link	5135	y
service	5141	n
track	5142	n
track_grade1	5143	n
track_grade2	5144	n
track_grade3	5145	n
track_grade4	5146	n
track_grade5	5147	n
bridleway	5151	n
cycleway	5152	n
footway	5153	n
path	5154	n
steps	5155	n
ferry	5160	n
unknown	5199	n

Supplementary Table 4. Railway types of OpenStreetMap data[20]

OSM railway types (RW)		
name (fclass)	**osm_code**	**included [y,n]**
rail	6101	y
light rail	6102	y
subway	6103	y
tram	6104	y
monorail	6105	n
narrow gauge	6106	n
miniature	6107	n
funicular	6108	n
rack	6109	n
drag lift	6111	n
chair lift	6112	n
cable car	6113	n
gondola	6114	n
goods	6115	n
other lift	6119	n

The conversion keys used to calculate areal extent of infrastructures with class-specific features widths are reported in Supplementary Table 5.

Supplementary Table 5. OSM class widths for the proxy area (INFRA) of the road and railway network (R and RW)

**OSM class**	**network**	**width [m]**
motorway (one way)	R	10
trunk	R	8
primary road	R	8
Secondary road	R	7
Tertiary road	R	6
Pedestrian	R	6
Living street	R	6
Unclassified roads	R	4.5
All railway classes	RW	5

3. Spatial indicators

In this section we describe the definition of the three groups of indicators in detail: a) built-up extent and pattern indicators, b) road network indicators and c) railway network indicators. The three groups of spatial indicators comprise different aspects of spatiality. Besides planar settlement data that describe the spatial configuration of urban areas, it is also important to include linear built-up data to consider road and public infrastructure networks between and within settlement areas. The first group of indicators primarily uses the vectorised settlement data derived from the LC100 grid. The second and third group of indicators rely on the filtered road and railway OSM data (Section 2).

Supplementary Tables 6-8 list all spatial indicators, followed by indicator equations and further technical and thematic descriptions. Please note that indicators whose equations use A_{REF} there exist two version, one referring to A_{NT} and the other to A_{IH}. All indicator values are freely available online[22].

Supplementary Table 6. Built-up land indicators

**Name**	**Abbreviation**	**Thematic description**	**Interpretation of values**	**Unit**
Fraction of built-up land	$\mathbf{BL_{fract}}$	BL_{fract} describes how much of a country's reference area is covered by built-up features.	High/low value: high/low fraction of the reference area covered with buildings	m^2/m^2
Built-up land per capita	$\mathbf{BL_{cap}}$	BL_{cap} quantifies the built-up area per capita in each country.	High/low value: high/low area of built-up land per capita	m^2/cap
Dispersion of built-up land	$\mathbf{BL_{disp}}$	BL_{disp} describes how spatially clustered the built-up land is.	High value: built-up land is dispersed<br>Low value: built-up land is clustered	no unit
Monocentricity of built-up land	$\mathbf{BL_{mono}}$	BL_{mono} discerns whether a country's built-up land is concentrated on one big urban center (monocentric) or dispersed on multiple similarly sized city centers (polycentric).	High value: monocentric<br>Low value: polycentric	m^2/m^2
Compactness of built-up land	$\mathbf{BL_{comp}}$	BL_{comp} indicates how "round" or "compact" the shape of BL in each country is (on average).	High value: round/compact patches of built-up land<br>Low value: irregular patches of built-up land	no unit
Urban population density	$\mathbf{UP_{dens}}$	UP_{dens} is defined as the number of people per unit area of urban built-up land, thereby indicating how closely people live together in cities.	High/low value: high/low population density in urban areas	cap / m^2

Supplementary Table 7. Road network indicators

Name	Abbreviation	Thematic description	Interpretation of values	Unit
Road density	**RD_{total}**	RD_{total} describes the density of a country's entire main road network.	High/low value: high/low density of roads per unit of inhabited land	m / m^2
Urban road density	**RD_{urban}**	RD_{urban} describes the density of roads in a country's urban areas.	High/low value: high/low density of roads in cities	m / m^2
Rural road density	**RD_{rural}**	RD_{rural} describes the density of a country's rural road network, indicating how "developed" (exploited) the countryside is. It is also a proxy for the connectivity between urban clusters.	High/low value: high/low density of roads in rural areas	m / m^2
Ratio of urban-to-rural road lengths	**$RL_{urb-rur}$**	$RL_{urb-rur}$ is the ratio of urban to rural road lengths in a country. If $RL_{urb-rur}$ is high, most roads are in urban areas	High value: low road connectivity between cities<br>Low value: high road connectivity between cities	no unit
Ratio of urban-to-rural road density	**$RD_{urb-rur}$**	The $RD_{urb-rur}$ is the ratio of RD_{urban} divided by RD_{rural}. Values indicate whether a country's road infrastructure is concentrated in urban or rural areas.	High value: road infrastructure development concentrated in urban areas<br>Low value: road infrastructure development concentrated in rural areas	no unit

Supplementary Table 8. Railway network indicators

Name	Abbreviation	Thematic description	Interpretation of values	Unit
Railway density	**RWD_{total}**	As RD_{total} but for railways	High/low value: high/low railway track density on IH	km / km^2
Urban railway density	**RWD_{urban}**	As RD_{urban} but for railways	High/low value: high/low railway track density in urban areas	km / km^2
Rural railway density	**RWD_{rural}**	As RD_{rural} but for railways	High/low value: high/low railway track density in rural areas	km / km^2
Ratio of urban-to-rural railway lengths	**$RWL_{urb-rural}$**	As $RL_{urb-rural}$ but for railways	High value: low railway connectivity between cities<br>Low value: high railway connectivity between cities	no unit
Ratio of urban-to-rural railway density	**$RWD_{urb-rur}$**	As $RD_{urb-rur}$ but for railways	High value: railway infrastructure development concentrated in urban areas<br>Low value: railway infrastructure development concentrated in rural areas	no unit

3.1 Built-up land indicators

The indicators for the extent and form of built-up area indicators are derived from the spatial information of the preprocessed BL-layers (Methods Section). Calculation of some indicators required population data, which we obtained from the World Bank[23].

3.1.1 Built-up land fraction (BL_{fract})

The Built-up land fraction (BL_{fract}) is defined as the area of built-up land (buildings and infrastructures, abbreviated as A_{BL} respectively A_{INFRA}) as percent of the area of the inhabited territory, abbreviated A_{IH} (unit: m^2/m^2).

Equation 1:

$$BL_{fract} = \frac{A_{BL} + A_{INFRA}}{A_{REF}}$$

3.1.2 Built-up land per capita (BL_{cap})

The BL_{cap} is defined as the area of buildings (A_{BL}) and infrastructures (A_{INFRA}) per inhabitant (POP_{total}) (unit: m^2/cap).

Equation 2:

$$BL_{cap} = \frac{A_{BL} + A_{INFRA}}{POP_{total}}$$

3.1.3 Dispersion of built-up land (BL_{disp})

The BL_{disp} describes the spatial clustering of built-up land and uses an Average Nearest Neighbor approach[24,25].

BL_{disp} is defined as the ratio between the average distance of each piece of built-up land to the nearest adjacent piece of built-up land and to the average distances by using a hypothetical random distribution. A high BL_{disp} value means that built-up land is dispersed (unit: m).

Equation 3:

$$BL_{disp} = \frac{D_O}{D_E}$$

$$D_O = \frac{\sum d_i}{n} \quad , \quad n = \frac{A_{BL}}{1km^2} \quad , \quad D_E = \frac{0.5}{\sqrt{\frac{n}{A_{REF}}}}$$

D_O … observed mean distance between each feature and its nearest neighbor

d_i … distance between feature *i* and its nearest neighboring feature

n … total number of features (1 feature per square meter A_{BL})

D_E … expected mean distance for the features given in a random pattern

3.1.4 Monocentricity of built-up land (BL_{mono})

The BL_{mono} is a measure of the monocentricity of built-up land, i.e. the size relations of a country's largest urban centers. Accordingly, we defined BL_{mono} as the area of the largest contiguous built-up feature as fraction of the area of the ten biggest contiguous built-up features in a country, as proposed in previous studies[26–28]. Low BL_{mono} values indicate polycentricity, high values dominance of one large center (unit: m^2/m^2).

Equation 4:

$$BL_{mono} = \frac{A_{BL-NR1}}{\sum A_{BL-TOP10}}$$

A_{BL-NR1} … a country's largest built-up feature (considering urban agglomerations, see section 2.1)

$\sum A_{BL-TOP10}$ … area sum of the ten biggest built-up features (considering urban agglomerations, see section 2.1)

3.1.5 Compactness of built-up land (BL_{comp})

In the 1960s and 1970s Cole, Gibbs and Richardson introduced many innovative compactness measurements to calculate urban compactness[29,30]. This indicator uses a physical form measurement method to derive a compactness measure of spatial shapes.[31]

The BL_{comp} measures how irregular the shape of built-up land is, compared to an area equivalent circle, which is perfectly round/compact. The BL_{comp} is area weighted. High BL_{comp} value indicates that built-up land approximates a circular shape (unitless index value).

Equation 5:

$$BL_{comp} = \frac{\sum_i (A_{BL\,i} * \frac{CFC_i}{CFBL_i})}{\sum A_{BL}}$$

$A_{BL\,i}$ ... area of a single BL-feature

CFC_i ... circumference of a circle with same area as single BL-feature

$CFBL_i$ ... circumference of single BL-feature

3.1.6 Urban population density (UP_{dens})

The UP_{dens} is the number of city dwellers per unit area of urban built-up land (unit: cap/m²), calculated for all urban areas of a country that are larger than 1 km².

Equation 6:

$$UP_{dens} = UPOP * \frac{POP_{total}}{\sum A_{BL \geq 1km^2}}$$

POP_{total} ... total population number

$UPOP$ ... urban population rate (see Supplementary Table 2)

$\sum A_{BL \geq 1km^2}$... area sum of built-up land feature bigger than 1km²

3.2 Road network indicators

The road (abbreviated R) network indicators are derived from the spatial information of the preprocessed BL-layers (Section 2.1) and filtered OSM data (Section 2.2).

3.2.1 Road density (RD_{total})

The RD_{total} is the length of roads per unit area of inhabited land (unit: m/m²); RL_{total} denotes the total road length of each country, and A_{IH} is the area of the inhabited land.

Equation 7:

$$RD_{total} = \frac{RL_{total}}{A_{REF}}$$

3.2.2 Urban road density (RD_{urban})

The RD_{urban} is the length of roads per unit area of urban land (unit: m/m²); RL_{urban} is the length of roads in urban areas, A_{BL} the area of a country's urban land.

Equation 8:

$$RD_{urban} = \frac{RL_{urban}}{A_{BL}}$$

3.2.3 Rural road density (RD_{rural})

The RD_{rural} is the length of roads per unit area of inhabited land (unit: m/m²); RL_{rural} is the length of roads in rural areas, which is calculated by subtracting RL_{urban} from RL_{total}. A_{REF} is a country's reference area and A_{BL} the area of a country's urban land.

Equation 9:

$$RD_{rural} = \frac{RL_{rural}}{A_{REF} - A_{BL}}$$

$$RL_{rural} = RL_{total} - RL_{urban}$$

3.2.4 Urban-rural road length ratio ($RL_{urb-rur}$)

The $RL_{urb-rur}$ is the ratio of the length of urban roads (RL_{urban}) divided by the length of rural roads (RL_{rural}). A high value of $RL_{urb-rur}$ indicates that a large fraction of road-kilometers is in urban land (unitless index value).

Equation 10:

$$RL_{urb-rur} = \frac{RL_{urban}}{RL_{rural}}$$

3.2.5 Urban-rural road density ratio ($RD_{urb-rur}$)

The $RD_{urb-rur}$ is the ratio of the RD_{urban} to RD_{rural}; variables as defined above (3.2.1. and 3.2.2). High $RD_{urb-rur}$ means that, in an international comparison, the concentration of roads in urban areas is higher than in other countries (unitless index value).

Equation 11:

$$RD_{urb-rur} = \frac{RD_{urban}}{RD_{rural}}$$

3.3 Railway network indicators

The railway (abbreviated RW) network indicators are derived from the spatial information of the preprocessed BL-layers (Section 2.1) and filtered OSM data (Section 2.2).

3.3.1 Railway density (RWD_{total})

The RWD_{total} is the density of a nation's railway track network per unit of national reference area. It is defined as total length of a country's railroad tracks per unit area of a country's reference area (unit: m/m^2); R_{WL} denotes railway length and A_{REF} the reference area.

Equation 12:

$$RWD_{total} = \frac{RWL_{total}}{A_{REF}}$$

3.3.2 Urban railway density (RWD_{urban})

The RWD_{urban} is the length of railroad track network in urban areas per unit area of urban land (unit: m/m^2); RWL_{urban} is the length of railway tracks in urban areas; A_{BL} is the area of a country's urban land.

Equation 13:

$$RWD_{urban} = \frac{RWL_{urban}}{A_{BL}}$$

3.3.3 Rural railway density (RWD_{rural})

The RWD_{rural} is the length of railway tracks per unit area of rural land in a nation (unit: m/m^2); abbreviations and definitions as for roads (Section 3.3.3).

Equation 14:

$$RWD_{rural} = \frac{RWL_{rural}}{A_{REF} - A_{BL}}$$

$$RWL_{rural} = RWL_{total} - RWL_{urban}$$

3.3.4 Urban-rural railway length ratio ($RWL_{urb\text{-}rur}$)

The $RWL_{urb\text{-}rur}$ is the ratio of the length of urban railways (RWL_{urban}) divided by the length of rural railways (RWL_{rural}). A high value of $RWL_{urb\text{-}rur}$ indicates that a large fraction of railway-kilometers is in urban land (unitless index value).

Equation 15:

$$RWL_{urb-rur} = \frac{RWL_{urban}}{RWL_{rural}}$$

3.3.5 Urban-rural railway density ratio ($\mathbf{RWD_{urb\text{-}rur}}$)

The $RWD_{urb-rur}$ is the ratio of the RWD_{urban} to RWD_{rural}; variables as defined above (3.3.1. and 3.3.2). High $RWD_{urb-rur}$ means that, in an international comparison, the concentration of railways in urban areas is higher than in other countries (unitless index value).

Equation 16:

$$RWD_{urb-rur} = \frac{RWD_{urban}}{RWD_{rural}}$$

4. Supplementary maps

The following Supplementary Figures 1-7 demonstrates important aspects of the spatial datasets underlying the indicators used in the analysis presented in the paper, in particular the indicators described in Table 1 in the main text.

Supplementary Figure 1 demonstrates the quality of various datasets to justify why we decided to use OSM data as basis for the analysis of built structures. Supplementary Figure 2 shows some examples of BL features of cities worldwide. Supplementary Figure 3 shows examples for infrastructures in cities as represented in our data. Supplementary Figure 4 demonstrates the added value of distinguishing urban and rural infrastructures. Supplementary Figure 5 shows global maps representing the built-up land indicators, Supplementary Figure 6 does the same for roads and Supplementary Figure 7 for railways.

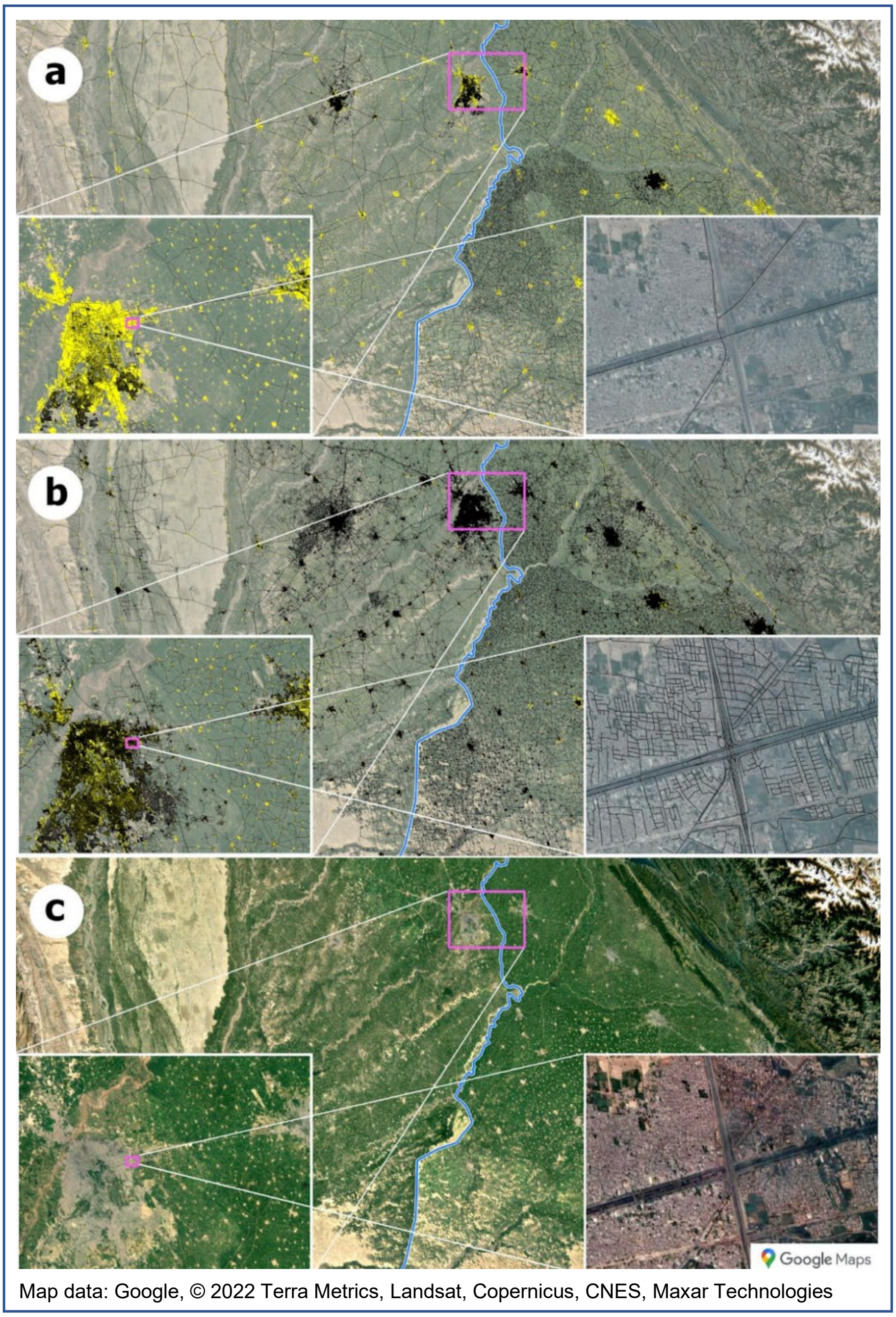

Supplementary Figure 1. Comparison of GRIP, Google satellite imagery and the OSM-derived dataset used in this study for a region at the Pakistani-Indian border near Lahore. Roads are shown in black, built-up areas (derived from LC100) in yellow. The blue line depicts the Pakistani-Indian border; we provide zooms to offer higher spatial detail. GRIP (a) and the filtered OSM data used in this study (b) show regional differences in data coverage. For comparison, map (c) provides the Google map image of the same area. While the rural road network is found to be denser in India than in Pakistan in both datasets, data quality varies much more in the GRIP dataset, as can be seen by comparing with Google map data (in particular, when looking at the zoom-in in the lower-right corner of part (c)). Data coverage within urban areas is clearly worse in the GRIP dataset compared to the filtered OSM data used in this study, as can be seen when looking at the translucent yellow patches indicating missing road data in densely occupied.

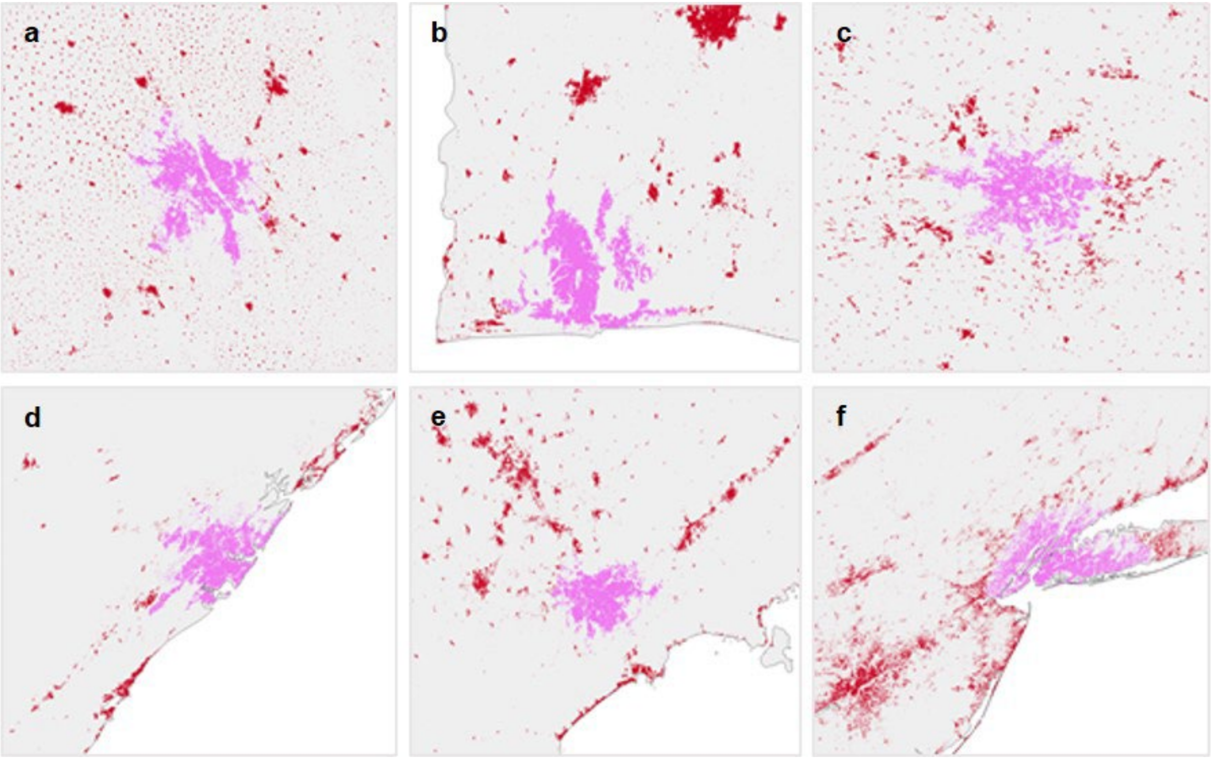

Supplementary Figure 2. Built-up land agglomerations. BL-agglomerations (pink) as clustered BL features (red) of (a) New Delhi, (b) Lagos, (c) Berlin, (d) Sydney, (e) São Paulo and (f) New York.

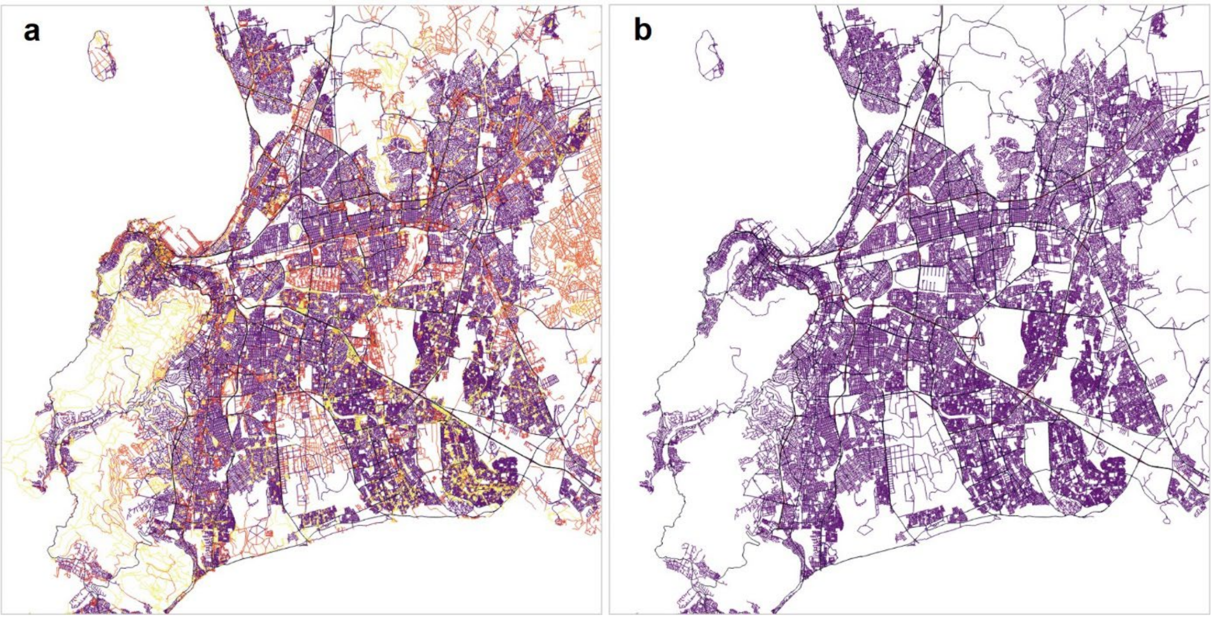

Supplementary Figure 3. OSM road network of Cape Town, South Africa. (a) Unfiltered and (b) filtered road network of Cape Town, South Africa. The unfiltered network shows all OSM classes, including minor roads (yellow to red lines) and high ranked roads (violet to dark blue lines). The filtered network shows only road data with an OSM-code smaller 5140, finally used for the spatial indicators. Copyright for administrative boundaries: © Eurogeographics

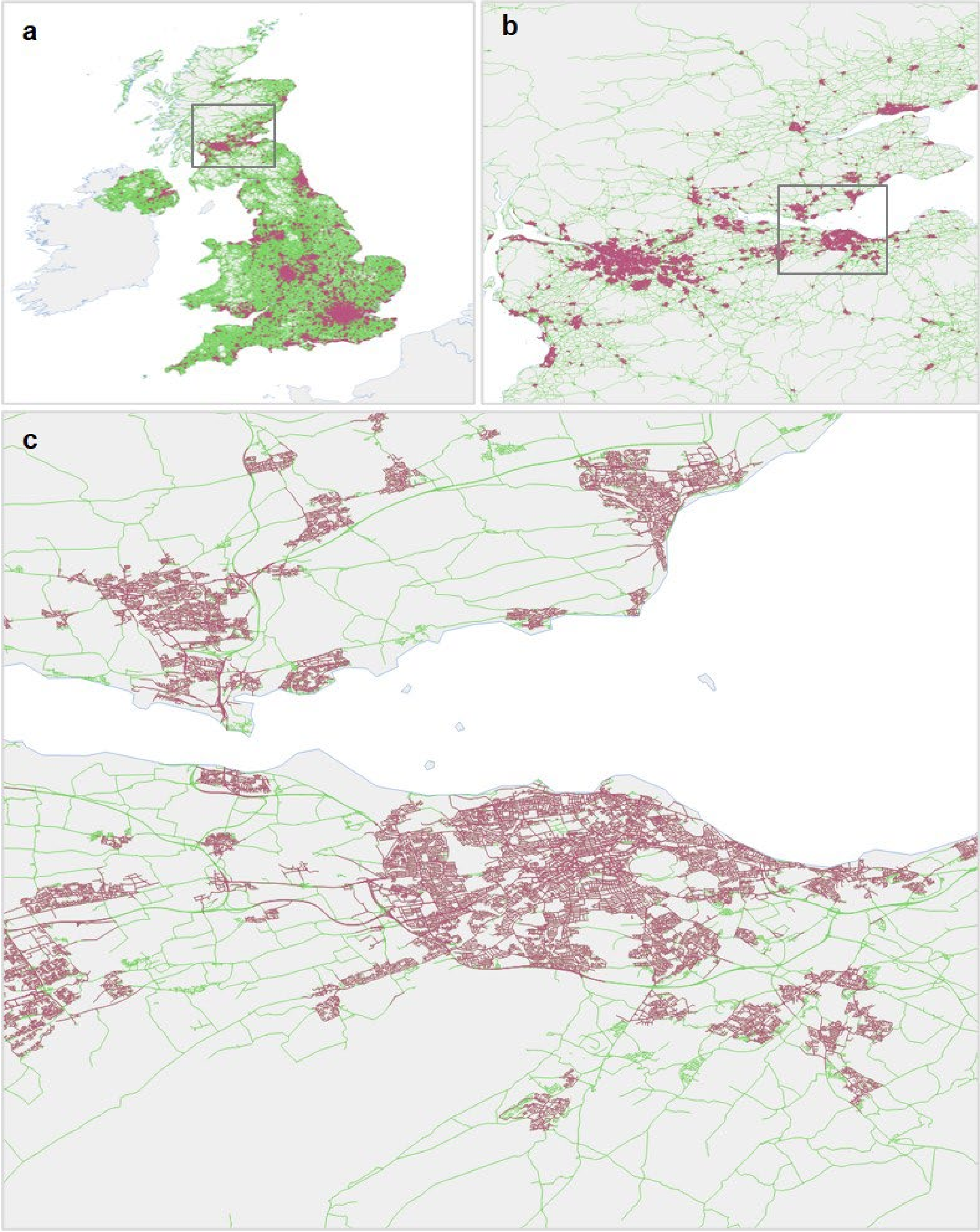

Supplementary Figure 4. Urban road network (red) and rural road network (green) of (a) the United Kingdom, (b) Central Scotland and (c) the city of Edinburgh in detail. Copyright for administrative boundaries: © Eurogeographics

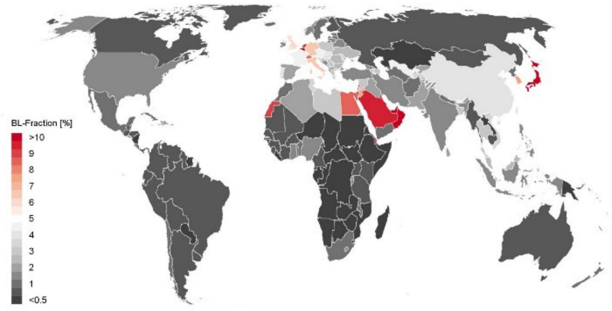

(a) Fraction of built-up land, BL_{fract}

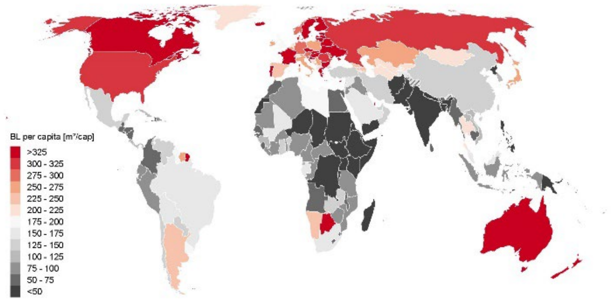

(b) Built-up land per capita, BL_{cap}

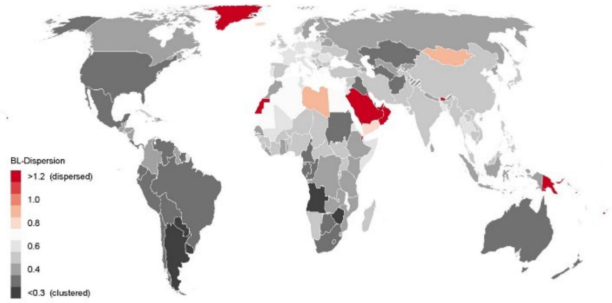

(c) Dispersion of built-up land, BL_{disp}

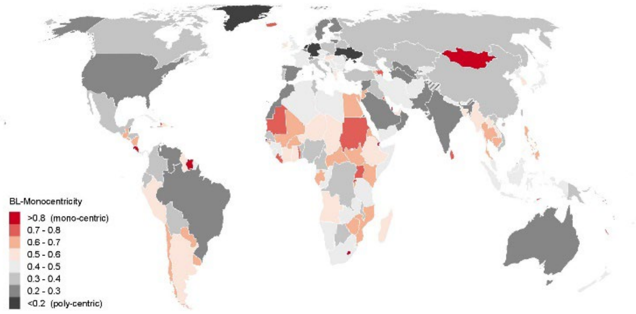

(d) Monocentricity of built-up land BL_{mono}

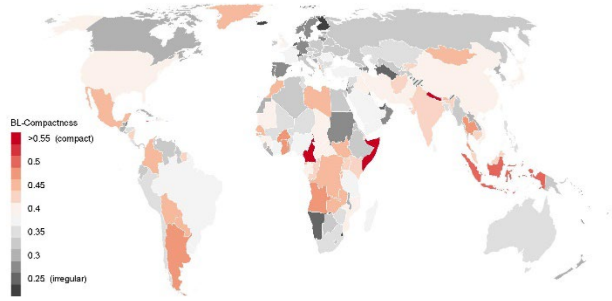

(e) Compactness of built-up land, BL_{comp}

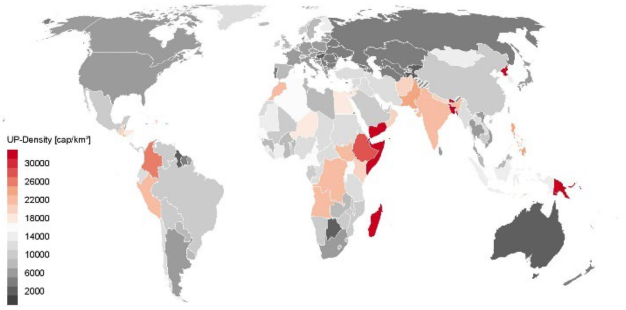

(f) Urban population density (UP_{dens})

Supplementary Figure 5. Maps representing the built-up land indicators. For explanation see the article text, in particular Table 1 in the article, as well the Supplementary Table 6. Copyright for administrative boundaries: © Eurogeographics

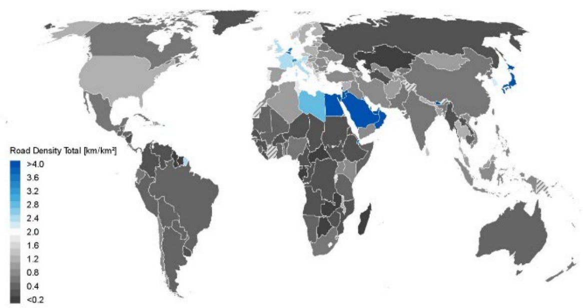

(a) Road density, RD_{total}

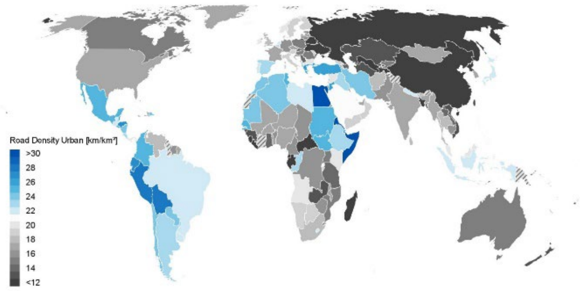

(b) Urban road density, RD_{urban}

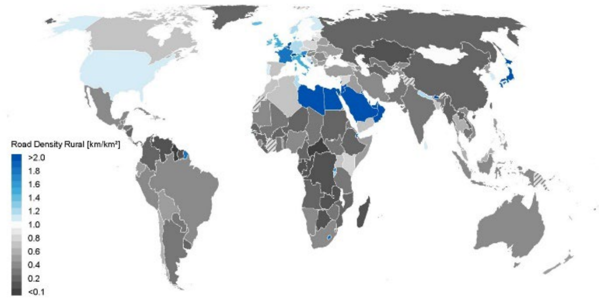

(c) Rural road density, RD_{rural}

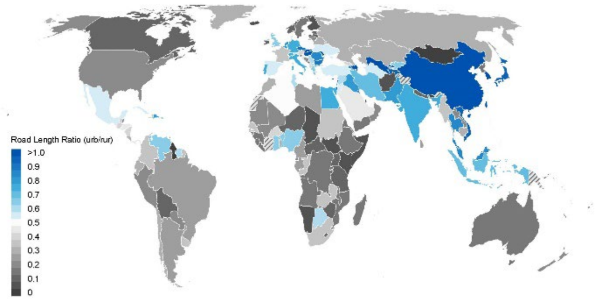

(e) Ratio of urban to rural road length $RL_{urb\text{-}rur}$

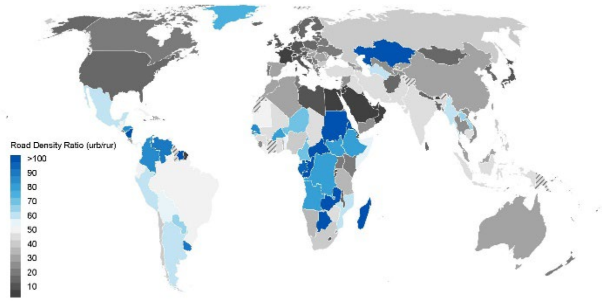

(f) Ratio of urban-to-rural road density $RD_{urb\text{-}rur}$

Supplementary Figure 6. Maps representing the road indicators. For explanation see the article text, in particular Table 1 in the article, as well the Supplementary Table 7. Copyright for administrative boundaries: © Eurogeographics

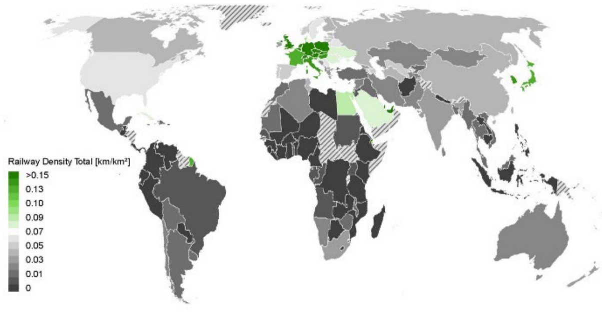

(a) Railway density, RD_{total}

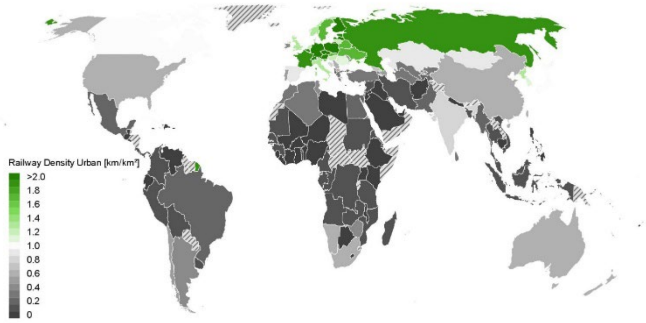

(b) Urban railway density, RD_{urban}

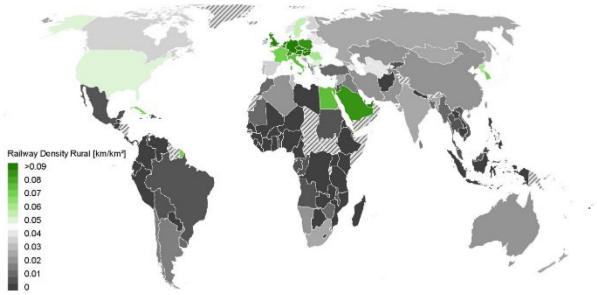

(c) Rural railway density, RD_{rural}

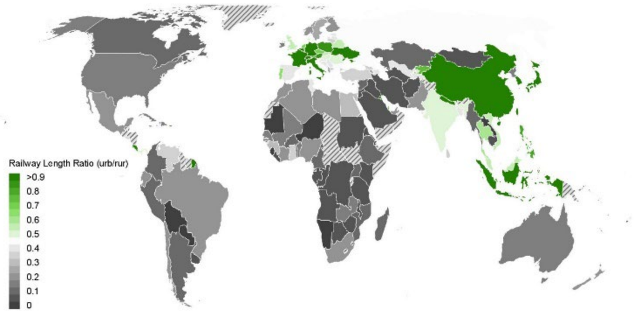

(e) Ratio of urban to rural railway length $RL_{urb-rur}$

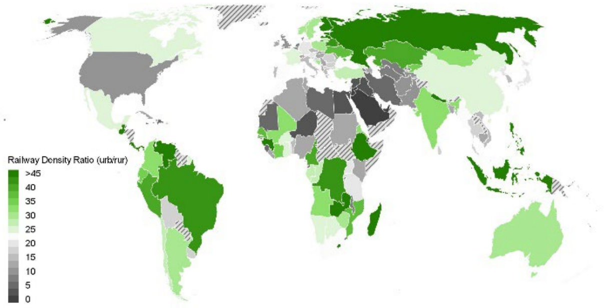

(f) Ratio of urban-to-rural railway density $RD_{urb-rur}$

Supplementary Figure 7. Maps representing the railway indicators. For explanation see the article text, in particular Table 1 in the article, as well the Supplementary Table 8. Copyright for administrative boundaries: © Eurogeographics

5. Additional analyses

5.1 Bivariate analyses using indicators related to total territory

Supplementary Table 9 shows results for indicators where total area of the national territory (A_{NT}) instead of inhabited land (A_{IH}) is used as reference area (A_{REF}) in all indicators that require a reference area (the others remain the same). The results are qualitatively similar to those using inhabited land as a reference; as those indicators that use A_{IH} as reference area have higher correlation coefficients than those using national territory, we included the former in the main paper.

Supplementary Table 9. Bivariate correlations using national territory as reference area

CO_2		TFC	
Indicator	Pearson's correlation coefficient	Indicator	Pearson's correlation coefficient
GDP	0.82	GDP	0.88
UPOP	0.72	BL_{cap}	0.72
BL_{cap}	0.69	UPOP	0.67
RWD_{urban}	0.56	RWD_{urb}	0.59
HDD	0.55	RWD_{total}	0.54
RWD_{total}	0.51	HDD	0.53
RWD_{rural}	0.50	RWD_{rural}	0.52
$RWL_{urb-rur}$	0.38	$RWL_{urb-rur}$	0.37
BL_{fract}	0.31	RD_{rural}	0.32
RD_{total}	0.29	RD_{total}	0.32
RD_{rural}	0.26	BL_{fract}	0.31
$RL_{urb-rur}$	0.19	BL_{disp}	0.12
BL_{disp}	0.08 (n.s.)	PGAS	0.12 (n.s.)
PGAS	0.01 (n.s.)	$RL_{urb-rur}$	0.08 (n.s.)
RD_{urban}	-0.05 (n.s.)	$RWD_{urb-rur}$	-0.05 (n.s.)
$RWD_{urb-rur}$	-0.05 (n.s.)	DENS	-0.10 (n.s.)
DENS	-0.08 (n.s.)	RD_{urban}	-0.11 (n.s.)
$RD_{urb-rur}$	-0.27	BL_{mono}	-0.31
BL_{mono}	-0.32	$RD_{urb-rur}$	-0.35
BL_{comp}	-0.36	BL_{comp}	-0.44
UP_{dens}	-0.43	UP_{dens}	-0.45

5.2 Alternative lasso approaches

Here we show that other procedures to choose the optimal model produce qualitatively similar results as the one presented in the main part of the article. We also included models predicting cross-country patterns of TFC and CO_2 exclusively from material stock pattern indicators (Models C). Supplementary Tables 10-12 compare Models A (all variables) and Models B (only conventional factors) with Models C that included only material stock pattern indicators. Models in Supplementary Table 10 were selected in the same manner as Table 2 in the main text, i.e. through cross-validation based on MSPE. Again, the same k=10-folds were used to assess all models; CVMSPE indicates the cross validation mean squared prediction error. Lasso paths for Models B and C are not shown; they are available from the authors on request. In Supplementary Table 11 shows the result of a lasso analysis in which the optimal models (λ^*) were derived by minimizing the BIC (Bayesian Information Criterion) instead of the MPSE. Supplementary Table 12 presents the results of an adaptive lasso analysis, where adaptive weights are used for penalizing different coefficients, and has been shown to provide a more consistent variable selection[32,33].

Supplementary Table 10. Lasso analysis using BIC to choose the penalty parameter

MODELS FOR TFC

LASSO PATH FOR MODEL A			ESTIMATED COEFFICIENTS			
**λ**	**(A)dded,(R)emoved**	**CVMSPE**	**Variable**	**MODEL A**	**MODEL B**	**MODEL C**
			GDP	0.494	0.572	
0.711	GDP(A)	0.703	DENS	-0.017	-0.039	
0.371	BL_{cap}(A)	0.319	UPOP			
0.161	HDD(A), RWD_{tot}(A)	0.179	HDD	0.021	0.051	
0.121	$RD_{urb-rur}$(A)	0.166	PGAS	-0.352	-0.288	
0.111	PGAS(A)	0.161	BL_{fract}			
0.101	RD_{urb}(A)	0.155	BL_{cap}	0.184		0.892
0.076	RWD_{urb}(A)	0.135	UP_{dens}			0.404
0.063	RWD_{tot}(R)	0.125	BL_{disp}			-0.236
0.048	DENS(A)	0.115	BL_{mono}	-0.017		-0.110
0.030	BL_{mono}(R)	0.108	BL_{comp}			-0.191
0.027	$RL_{urb-rur}$(A)	0.107	RD_{tot}			
**0.017***	**Unchanged**	**0.105**	RD_{urb}	-0.392		
0.016	$RWD_{urb-rur}$(A)	0.105	RD_{rur}			
0.013	BL_{comp}(A)	0.105	$RL_{urb-rur}$			
0.011	(Unchanged)	0.106	$RD_{urb-rur}$	-0.074		-0.311
			RWD_{tot}			0.110
			RWD_{urb}	0.045		
			RWD_{rur}			
			$RWL_{urb-rur}$			
			$RWD_{urb-rur}$			
			Intercept	3.457	2.542	-2.867

**Measures of in-and-out-of-sample fit**			
BIC	84.17	100.32	183.788
r^2	0.900	0.851	0.727
oSr^2	0.865	0.833	0.654

MODEL FOR CO_2

LASSO PATH FOR MODEL A			ESTIMATED COEFFICIENTS			
**λ**	**(A)dded,(R)emoved**	**CVMSPE**	**Variable**	**MODEL A**	**MODEL B**	**MODEL C**
0.867	GDP(A)	1.278	GDP	0.449	0.582	
0.544	BL_{cap}(A)	0.801	DENS	-0.061	0.023	
0.496	UPOP(A)	0.738	UPOP	0.466	0.558	
0.452	RWD_{tot}(A)	0.683	HDD	0.055	0.109	
0.312	HDD(A)	0.530	PGAS	-0.680	-0.688	
0.215	PGAS(A)	0.446	BL_{fract}	0.201		0.295
0.135	$RD_{urb-rur}$(A)	0.338	BL_{cap}	0.176		1.294
0.123	BL_{fract}(A)	0.323	UP_{dens}			0.727
0.085	$RWL_{urb-rur}$(A)	0.289	BL_{disp}			-0.453
0.070	BL_{fract}(R)	0.280	BL_{mono}			-0.207
0.048	BL_{comp}(A)	0.269	BL_{comp}	0.489		0.194
0.044	RD_{urb}(A)	0.266	RD_{tot}			
0.037	RWD_{urb}(A)	0.262	RD_{urb}	-0.201		
0.025	BL_{fract} (A)	0.256	RD_{rur}			0.118
0.023	DENS(A)	0.255	$RL_{urb-rur}$			
0.021	$RWL_{urb-rur}$(R)	0.254	$RD_{urb-rur}$			
0.016	RWD_{rur}(A)	0.251	RWD_{tot}			
0.016	$RD_{urb-rur}$(R)	0.251	RWD_{urb}	0.130		
0.014	RWD_{tot}(R)	0.250	RWD_{rur}	0.017		0.158
**0.012***	**Unchanged**	**0.249**	$RWL_{urb-rur}$			
0.006	Unchanged	0.250	$RWD_{urb-rur}$			-0.045
			Intercept	-2.723	-4.294	-12.018

**Measures of in-and-out-of-sample fit**			
BIC	178.44	190.82	260.192
r^2	0.873	0.812	0.716
oSr^2	0.817	0.785	0.618

Supplementary Table 11. Lasso analysis using BIC to choose the penalty parameter

MODELS FOR TFC

LASSO PATH FOR MODEL A			ESTIMATED COEFFICIENTS			
**λ**	**(A)dded,(R)emoved**	**BIC**	**Variable**	**MODEL A**	**MODEL B**	**MODEL C**
			GDP	0.480	0.581	
0.711	GDP(A)	285.728	DENS	-0.017	-0.043	
0.371	BL_{cap}(A)	200.124	UPOP			
0.161	HDD(A), RWD_{tot}(A)	141.539	HDD	0.020	0.054	
0.121	$RD_{urb-rur}$(A)	134.325	PGAS	-0.296	-0.315	
0.111	PGAS(A)	131.781	BL_{fract}			
0.101	RD_{urb}(A)	128.450	BL_{cap}	0.187		0.892
0.076	RWD_{urb}(A)	112.658	UP_{dens}			0.404
0.063	RWD_{tot}(R)	97.342	BL_{disp}			-0.236
0.048	DENS(A)	91.507	BL_{mono}	-0.017		-0.110
**0.030***	**BL_{mono}(R)**	**82.064**	BL_{comp}			-0.191
0.027	$RL_{urb-rur}$(A)	85.258	RD_{tot}			
			RD_{urb}	-0.324		
			RD_{rur}			
			$RL_{urb-rur}$			
			$RD_{urb-rur}$	-0.064		-0.311
			RWD_{tot}			0.110
			RWD_{urb}	0.039		
			RWD_{rur}			
			$RWL_{urb-rur}$			
			$RWD_{urb-rur}$			
			Intercept	3.076	2.533	-2.867

**Measures of in-and-out-of-sample fit**			
BIC	82.06	99.66	183.788
r^2	0.893	0.853	0.727
oSr^2	0.860	0.832	0.654

MODELS FOR CO_2

LASSO PATH FOR MODEL A			ESTIMATED COEFFICIENTS			
**λ**	**(A)dded,(R)emoved**	**BIC**	**Variable**	**MODEL A**	**MODEL B**	**MODEL C**
0.867	GDP(A)	351.072	GDP	0.449	0.572	
0.544	BL_{cap}(A)	301.531	DENS	-0.061		
0.496	UPOP(A)	295.699	UPOP	0.466	0.542	
0.452	RWD_{tot}(A)	290.575	HDD	0.055	0.101	
0.312	HDD(A)	261.447	PGAS	-0.680	-0.613	
0.215	PGAS(A)	240.400	BL_{fract}	0.201		0.193
0.135	$RD_{urb-rur}$(A)	207.087	BL_{cap}	0.176		1.076
0.123	BL_{fract}(A)	206.571	UP_{dens}			0.464
0.085	$RWL_{urb-rur}$(A)	196.252	BL_{disp}			-0.108
0.070	BL_{fract}(R)	186.098	BL_{mono}			-0.181
0.048	BL_{comp}(A)	183.987	BL_{comp}	0.489		
0.044	RD_{urb}(A)	186.758	RD_{tot}			
0.037	RWD_{urb}(A)	188.144	RD_{urb}	-0.201		
0.025	BL_{fract}	188.701	RD_{rur}			0.150
0.023	DENS(A)	192.327	$RL_{urb-rur}$			
0.021	$RWL_{urb-rur}$(R)	186.487	$RD_{urb-rur}$			
0.016	RWD_{rur}(A)	184.334	RWD_{tot}			
0.016	$RD_{urb-rur}$(R)	184.334	RWD_{urb}	0.130		
0.014	RWD_{tot}(R)	179.105	RWD_{rur}	0.017		0.167
**0.012***	**(Unchanged)**	**177.299**	$RWL_{urb-rur}$			
0.006	(Unchanged)	181.956	$RWD_{urb-rur}$			-
			Intercept	-2.723	-4.074	-8.624

**Measures of in-and-out-of-sample fit**			
BIC	178.44	186.94	258.453
r^2	0.873	0.817	0.697
oSr^2	0.817	0.786	0.610

Supplementary Table 12. Model selection using the adaptive lasso cross-validation approach

MODELS FOR TFC

**LASSO PATH FOR MODEL A**			**ESTIMATED COEFFICIENTS**			
**λ**	**(A)dded,(R)emoved**	**CVMSPE**	**Variable**	**MODEL A**	**MODEL B**	**MODEL C**
			GDP	0.480	0.581	
0.711	GDP(A)	285.728	DENS	-0.017	-0.043	
0.371	BL_{cap}(A)	200.124	UPOP			
0.161	HDD(A), RWD_{tot}(A)	141.539	HDD	0.020	0.054	
0.121	$RD_{urb-rur}$(A)	134.325	PGAS	-0.296	-0.315	
0.111	PGAS(A)	131.781	BL_{fract}			
0.101	RD_{urb}(A)	128.450	BL_{cap}	0.187		1.015
0.076	RWD_{urb}(A)	112.658	UP_{dens}			0.518
0.063	RWD_{tot}(R)	97.342	BL_{disp}			-0.208
0.048	DENS(A)	91.507	BL_{mono}	-0.017		
**0.030***	**BL_{mono}(R)**	**82.064**	BL_{comp}			
0.027	$RL_{urb-rur}$(A)	85.258	RD_{tot}			
			RD_{urb}	-0.324		
			RD_{rur}			
			$RL_{urb-rur}$			
			$RD_{urb-rur}$	-0.064		-0.301
			RWD_{tot}			0.119
			RWD_{urb}	0.039		
			RWD_{rur}			
			$RWL_{urb-rur}$			
			$RWD_{urb-rur}$			
			Intercept	3.076	2.533	-4.719

**Measures of in-and-out-of-sample fit**			
BIC	82.06	99.66	174.70
r^2	0.893	0.853	0.725
oSr^2	0.860	0.832	0.687

MODELS FOR CO_2

**LASSO PATH FOR MODEL A**			**ESTIMATED COEFFICIENTS**			
**λ**	**(A)dded,(R)emoved**	**CVMSPE**	**Variable**	**MODEL A**	**MODEL B**	**MODEL C**
5.448	GDP(A)	1.278	GDP	0.470	0.589	
0.847	PGAS(A)	0.506	DENS			
0.485	HDD(A)	0.431	UPOP	0.400	0.529	
0.403	RWD_{urb}(A)	0.395	HDD	0.055	0.105	
0.253	UPOP(A),BL_{fract}(A)	0.329	PGAS	-0.773	-0.661	
0.230	BL_{cap}(A)	0.318	BL_{fract}	0.139		0.273
0.063	BL_{comp}(A)	0.240	BL_{cap}	0.227		1.356
**0.030***	**Unchanged**	**0.230**	UP_{dens}			0.736
0.025	RD_{urb}(A)	0.231	BL_{disp}			
0.021	DENS(A)	0.231	BL_{mono}			
0.002	Unchanged	0.230	BL_{comp}	0.308		
			RD_{tot}			
			RD_{urb}			
			RD_{rur}			
			$RL_{urb-rur}$			
			$RD_{urb-rur}$			
			RWD_{tot}			
			RWD_{urb}	0.145		
			RWD_{rur}			0.141
			$RWL_{urb-rur}$			
			$RWD_{urb-rur}$			
			Intercept	-3.774	-4.067	-13.315

**Measures of in-and-out-of-sample fit**			
BIC	169.09	186.48	243.97
r^2	0.867	0.817	0.697
oSr^2	0.830	0.789	0.660

5.3 Alternative variable section using forward stepwise regressions

We also used forward stepwise regressions to select variables in a multiple regression model (Supplementary Table 13). We established a linear model from the whole set of conventional factors and material stock pattern indicators to obtain a parsimonious model. The method begins with an initial constant model and searches for terms to add to the model or remove from the model taking forward or backward steps. The criterion for inclusion respectively exclusion is based on the p-value of an F-statistic to test models with and without the potential term at each step. A term is removed from the model if there is insufficient evidence to reject the null hypothesis that the term has a zero coefficient. Conversely, a term currently not in the model is added if there is evidence to reject the hypothesis that the term would have a zero coefficient if included. We used 10% significance as criterion for exclusion and 5% for inclusion. The same 10-folds were used as in the lasso analyses. We also evaluated the performance of the selected models (Models A) in both in-sample and (cross-validation) out-of-sample fitting. Once more, the results are compared to a benchmark Models B, in which selection considered only conventional factors, and Models C that included only material stock pattern indicators (selection paths for other models not shown; available on request). Again, BL_{cap} is always selected in Models A, along with other material stock pattern indicators, and Models A outperform Models B, which are better than Models C. Alternative criterion rules to add or remove terms (e.g., using BIC instead of the F-statistic) gave similar results (available from the authors for non-commercial research purposes upon reasonable request).

Supplementary Table 13. Model selection using forward stepwise regression

MODELS FOR TFC

STEPWISE PATH FOR MODEL A		ESTIMATED COEFFICIENTS			
**(A)dded,(R)emoved**	**pval**	**Variable**	**MODEL A**	**MODEL B**	**MODEL C**
GDPCAP(A)	0.000	GDP	0.546	0.582	
BL_{cap}(A)	0.000	DENS			
PGAS(A)	0.000	UPOP			
RD_{urb}(A)	0.000	HDD	0.038	0.060	
HDD(A)	0.001	PGAS	-0.401	-0.370	
		BL_{fract}			
		BL_{cap}	0.194		1.024
		UP_{dens}			0.521
		BL_{disp}			
		BL_{mono}	-0.017		
		BL_{comp}			
		RD_{tot}			
		RD_{urb}	-0.559		
		RD_{rur}			0.205
		$RL_{urb-rur}$			
		$RD_{urb-rur}$			
		RWD_{tot}			0.141
		RWD_{urb}			
		RWD_{rur}			
		$RWL_{urb-rur}$			
		$RWD_{urb-rur}$			
		Intercept	3.185	2.318	-5.497

**Measures of in-and-out-of-sample fit**			
BIC	67.50	97.75	175.23
r^2	0.894	0.849	0.712
oSr^2	0.876	0.836	0.668

MODEL FOR CO2

STEPWISE PATH FOR MODEL A		ESTIMATED COEFFICIENTS			
**(A)dded,(R)emoved**	**pval**	**Variable**	**MODEL A**	**MODEL B**	**MODEL C**
GDP(A)	0.000	GDP	0.476	0.586	
PGAS(A)	0.000	DENS			
HDD(A)	0.000	UPOP	0.408	0.546	
RWD_{tot}(A)	0.000	HDD	0.063	0.106	
BL_{cap}(A)	0.000	PGAS	-0.753	-0.667	
BL_{comp}(A)	0.001	BL_{fract}			
UPOP(A)	0.016	BL_{cap}	0.258		1.302
		UP_{dens}			0.715
		BL_{disp}			
		BL_{mono}			
		BL_{comp}	0.680		
		RD_{tot}			0.283
		RD_{urb}			
		RD_{rur}			
		$RL_{urb-rur}$			
		$RD_{urb-rur}$			
		RWD_{tot}	0.184		0.197
		RWD_{urb}			
		RWD_{rur}			
		$RWL_{urb-rur}$			
		$RWD_{urb-rur}$			
		Intercept	-3.031	-4.141	-12.508

**Measures of in-and-out-of-sample fit**			
BIC	166.37	186.46	245.683
r^2	0.865	0.818	0.692
oSr^2	0.830	0.787	0.648

Data and Code availability

Datasets on spatial data on patterns of global infrastructure and settlements, the inhabited land layer, as well as the indicator values of dependent and independent variables used in the statistical analyses is freely available here: https://doi.org/10.5281/zenodo.5876941. Code used for calculations of maps is freely available here: https://doi.org/10.5281/zenodo.5883652. An interim result that was too large to be uploaded as zenodo archive is available from the authors for non-commercial research purposes upon reasonable request (for detail see[60]).

Supplementary References

1. Corbane, C., Sabo, F., Politis, P. & Vasileos, S. *GHS-BUILT-S2 R2020A - built-up grid derived from Sentinel-2 global image composite for reference year 2018 using Convolutional Neural Networks (GHS-S2Net)*. (Joint Research Centre (JRC), 2020).
2. Esch, T. *et al.* Breaking new ground in mapping human settlements from space – The Global Urban Footprint. *ISPRS Journal of Photogrammetry and Remote Sensing* **134**, 30–42 (2017).
3. Gong, P. *et al.* Annual maps of global artificial impervious area (GAIA) between 1985 and 2018. *Remote Sensing of Environment* **236**, 111510 (2020).
4. Marcel Buchhorn *et al.* Copernicus Global Land Service: Land Cover 100m: collection 2: epoch 2015: Globe. (2019) doi:10.5281/zenodo.3243509.
5. Buchhorn, M. *et al.* Copernicus Global Land Cover Layers—Collection 2. *Remote Sensing* **12**, 1044 (2020).
6. Tsendbazar, N. *et al.* Towards operational validation of annual global land cover maps. *Remote Sensing of Environment* **266**, 112686 (2021).
7. Meijer, J. R., Huijbregts, M. A. J., Schotten, K. C. G. J. & Schipper, A. M. Global patterns of current and future road infrastructure. *Environ. Res. Lett.* **13**, 064006 (2018).
8. Jiang, B. & Jia, T. Zipf's law for all the natural cities in the United States: a geospatial perspective. *International Journal of Geographical Information Science* **25**, 1269–1281 (2011).
9. Haklay, M. How Good is Volunteered Geographical Information? A Comparative Study of OpenStreetMap and Ordnance Survey Datasets. *Environ Plann B Plann Des* **37**, 682–703 (2010).
10. Cao, W., Dong, L., Wu, L. & Liu, Y. Quantifying urban areas with multi-source data based on percolation theory. *Remote Sensing of Environment* **241**, 111730 (2020).
11. Barrington-Leigh, C. & Millard-Ball, A. The world's user-generated road map is more than 80% complete. *PLOS ONE* **12**, e0180698 (2017).
12. Yan, Y. *et al.* Volunteered geographic information research in the first decade: a narrative review of selected journal articles in GIScience. *International Journal of Geographical Information Science* **34**, 1765–1791 (2020).
13. Sikder, S. K., Nagarajan, M., Kar, S. & Koetter, T. A geospatial approach of downscaling urban energy consumption density in mega-city Dhaka, Bangladesh. *Urban Climate* **26**, 10–30 (2018).
14. Hecht, R., Kunze, C. & Hahmann, S. Measuring Completeness of Building Footprints in OpenStreetMap over Space and Time. *ISPRS International Journal of Geo-Information* **2**, 1066–1091 (2013).
15. Boeing, G. Urban spatial order: street network orientation, configuration, and entropy. *Appl Netw Sci* **4**, 1–19 (2019).
16. Barrington-Leigh, C. & Millard-Ball, A. Global trends toward urban street-network sprawl. *PNAS* **117**, 1941–1950 (2020).

17. Goodchild, M. F. & Li, L. Assuring the quality of volunteered geographic information. *Spatial Statistics* **1**, 110–120 (2012).

18. Löw, M. & Matej, S. *Software code to calculate datasets on global patterns of settlements and infrastructures*. (https://doi.org/10.5281/zenodo.5883652, 2023).

19. NOAA National Centers for Environmental Information. Global Land One-km Base Elevation Project (GLOBE Topography). https://www.ngdc.noaa.gov/mgg/topo/globe.html.

20. Geofabrik GmbH. OpenStreetMap Data Extracts - Geofabrik Download Server. https://download.geofabrik.de/.

21. Eurostat. Countries - GISCO archive. https://ec.europa.eu/eurostat/web/gisco/geodata/reference-data/administrative-units-statistical-units/countries.

22. Löw, M. *et al. Datasets on global patterns of settlements and infrastructures*. (https://doi.org/10.5281/zenodo.5876941, 2023).

23. World Bank Group. Total Population - World Bank Data. https://data.worldbank.org/indicator/SP.POP.TOTL.

24. Ebdon, D. *Statistics in Geography*. (Blackwell Publishing (2nd edition), 1985).

25. ESRI. *How Average Nearest Neighbor works*. (ArcGIS Pro 2.7, 2021).

26. ESPON. *Polycentric Urban Development and Rural-Urban Partnership - Thematic Study of INTERREG and ESPON activities*. (Report of the European Spatial Planning Observation Network (ESPON), Coordination Unit, 2004).

27. Meijers, E. J. & Burger, M. J. Spatial Structure and Productivity in US Metropolitan Areas. *Environ Plan A* **42**, 1383–1402 (2010).

28. Parr, J. The Polycentric Urban Region: A Closer Inspection. *Regional Studies* **38**, 231–240 (2004).

29. Cole, J. P. *Study of Major and Minor Civil Division*. (Political Geography; Mimeographed, 1960).

30. Gibbs, J. P. *Urban Research Methods*. (Van Nostrand, 1961).

31. Richardson, H. W. *The economics of urban size*. (Saxon House, 1973).

32. Zou, H. The adaptive lasso and its oracle properties. *Journal of the American Statistical Association* **101**, 1418–1429 (2006).

33. Zhang, Y., Li, R. & Tsai, C. L. Regularization parameter selections via generalized information criterion. *Journal of the American Statistical Association* **105**, 312–323 (2010).
